# The role of sensorimotor experience in the development of mimicry in infancy

**DOI:** 10.1111/desc.12771

**Published:** 2018-12-04

**Authors:** Carina C. J. M. de Klerk, Iona Lamy‐Yang, Victoria Southgate

**Affiliations:** ^1^ Centre for Brain and Cognitive Development Birkbeck College University of London London UK; ^2^ School of Psychology Cardiff University Wales UK; ^3^ Department of Psychology University of Copenhagen Copenhagen Denmark

**Keywords:** associative learning, EMG, infancy, mimicry, parent‐child interaction

## Abstract

During social interactions we often have an automatic and unconscious tendency to copy or ‘mimic’ others’ actions. The dominant view on the neural basis of mimicry appeals to an automatic coupling between perception and action. It has been suggested that this coupling is formed through associative learning during correlated sensorimotor experience. Although studies with adult participants have provided support for this hypothesis, little is known about the role of sensorimotor experience in supporting the development of perceptual‐motor couplings, and consequently mimicry behaviour, in infancy. Here we investigated whether the extent to which an observed action elicits mimicry depends on the opportunity an infant has had to develop perceptual‐motor couplings for this action through correlated sensorimotor experience. We found that mothers’ tendency to imitate their 4‐month‐olds’ facial expressions during a parent‐child interaction session was related to infants’ facial mimicry as measured by electromyography. Maternal facial imitation was not related to infants’ mimicry of hand actions, and instead we found preliminary evidence that infants’ tendency to look at their own hands may be related to their tendency to mimic hand actions. These results are consistent with the idea that mimicry is supported by perceptual‐motor couplings that are formed through correlated sensorimotor experience obtained by observing one's own actions and imitative social partners.

**RESEARCH HIGHLIGHTS**

- Mimicry, the tendency to spontaneously and unconsciously copy others’ actions, plays an important role in social interactions, yet little is known about its development.
- It has been suggested that mimicry is supported by perceptual‐motor couplings that develop through associative learning during correlated sensorimotor experience.
- We investigated whether the extent to which an action elicits mimicry relates to the opportunities infants have had to develop perceptual‐motor couplings for this action.
- Mothers’ tendency to imitate their 4‐month‐olds’ facial actions related to infants’ facial mimicry; preliminary evidence suggests that infants’ interest in their own hands related to their hand mimicry.

## 1 INTRODUCTION

Mimicry, the spontaneous and unconscious tendency to copy others’ behaviour, is ubiquitous in our everyday social interactions. In the past decades, a wealth of research has demonstrated that mimicry plays an important role in communication and affiliation, for example, by enhancing liking and rapport, and by increasing the smoothness of social interactions (for a review see Chartrand & Lakin, 2013). However, despite the important social functions that mimicry is thought to serve, little is known about its ontogeny. Mimicry is thought to be supported by the mirror neuron system (MNS), and more specifically by connections between the superior temporal sulcus (STS), involved in processing the kinematics of observed actions, and the inferior frontal gyrus (IFG), that represents the motor commands needed to perform these actions (Likowski, Mühlberger, Gerdes, Wieser, Pauli, & Weyers, 2012; Wang, Ramsey, & Hamilton, 2011). These connections provide a direct link between perception and action, where the perception of an action activates the motor representation of this action, and are thought to play a crucial role in the implementation of mimicry and other automatic visual‐motor responses (e.g. Bien, Roebroeck, Goebel, & Sack, 2009; Heyes, 2011; Iacoboni, Woods, Brass, Bekkering, Mazziotta, & Rizzolatti, 1999). Indeed, it has been shown that connectivity between STS and IFG increases when participants perform an automatic imitation task (Wang et al., 2011) and that IFG activation during the observation of facial expressions correlates with facial mimicry responses (Likowski et al., 2012).

One of the most popular views with regard to the ontogeny of the MNS is that this coupling between “seeing” and “doing” is inborn (Bertenthal & Longo, 2007; Lepage & Théoret, 2007; Meltzoff & Decety, 2003; Rizzolatti, Fadiga, Fogassi, & Gallese, 2002) and that it supports infants’ ability to copy facial actions from birth (Meltzoff & Moore, 1977; Simpson, Murray, Paukner, & Ferrari, 2014). However, the reports of neonatal imitation of facial actions have been subject to extensive criticism and debate (e.g. Jones, 2009; Meltzoff, Murray, Simpson, Heimann, Nagy, Nadel, & Subiaul, 2017; Oostenbroek, Redshaw, Davis, Kennedy‐Costantini, Nielsen, Slaughter, & Suddendorf, 2018; Oostenbroek, Suddendorf, Nielsen, Redshaw, Kennedy‐Costantini, Davis, & Slaughter, 2016; Ray & Heyes, 2011), and in recent years evidence has been accumulating for alternative accounts that suggest that couplings between visual and motor representations of actions instead develop as a result of associative learning during correlated sensorimotor experience (Cook, Bird, Catmur, Press, & Heyes, 2014; Heyes, 2001; Keysers & Perrett, 2004). Support for these *sensorimotor learning* accounts has been provided by studies demonstrating that in adults, correlated sensorimotor experience can enhance (Press, Gillmeister, & Heyes, 2007), abolish (Heyes, Bird, Johnson, & Haggard, 2005), reverse (Catmur, Walsh, & Heyes, 2007), or induce (Landmann, Landi, Grafton, & Della‐Maggiore, 2011) perceptual–motor couplings. Furthermore, recent studies have provided the first evidence that associative learning may also underlie the initial formation of perceptual‐motor couplings in the developing brain (e.g. de Klerk, Johnson, Heyes, & Southgate, 2015; O'Sullivan, Bijvoet‐van den Berg, & Caldwell, 2018).

If perceptual‐motor couplings are required for mimicry, and if these couplings indeed develop through associative learning, then the extent to which an observed action will elicit mimicry should depend on the extent to which the infant has experienced repeated visuo‐motor contingency with this action. For *perceptually transparent* actions, such as hand and arm actions, this experience can be obtained through self‐observation. For example, 2‐ to 3‐month‐old infants have been found to spend much of the time that they are awake observing their own hands (White, Castle, & Held, 1964) and newborn infants appear to purposely control their arm movements to keep their hands visible (van der Meer, 1997; van der Meer, van der Weel, & Lee, 1995). Although this visual preference for their own hands most likely serves a function in increasing motor control (von Hofsten, 2004), it also provides infants with plenty of opportunities to develop perceptual‐motor couplings for arm and hand actions (Del Giudice, Manera, & Keysers, 2009). However, *for perceptually opaque* actions, such as facial actions, which infants cannot observe themselves perform, visual feedback from imitative social partners is hypothesized to provide the necessary input to form perceptual‐motor couplings (Heyes, 2010; Ray & Heyes, 2011). Infants have an early preference for faces (Fantz, 1963; Morton & Johnson, 1991; Valenza, Simion, Cassia, & Umiltà, 1996) and spend a large proportion of their awake time in face‐to‐face interactions with their caregivers. As these interactions contain frequent imitative episodes (Jones, 2009; Moran, Krupka, Tutton, & Symons, 1987; Pawlby, 1977), the correlated visuomotor experience that infants receive during these interactions may support the development of perceptual‐motor couplings for facial actions, which in turn allow the infant to mimic these actions. In line with this idea, a recent study found that maternal imitation of infants’ facial expressions at 2 months related to infants’ sensorimotor cortex activation as measured by EEG during the observation of the same facial expressions at 9 months (Rayson, Bonaiuto, Ferrari, & Murray, 2017). Although this sensorimotor cortex activation is thought to reflect the existence of perceptual‐motor couplings which give rise to mimicry ‐ as it can be measured both during the execution and observation of actions (e.g. Pineda, 2005; Southgate, Johnson, Osborne, & Csibra, 2009), this study did not investigate how the activation of the sensorimotor cortex during the observation of facial actions actually related to the infants’ ability to copy those actions.

This study aimed to fill this gap by investigating whether infants who receive greater amounts of maternal facial imitation show a greater tendency to mimic those specific actions when performed by others. To further assess the specificity of this relationship, we also investigated the relationship between maternal imitation and infants’ mimicry of perceptually transparent hand actions. As parental imitation is infants’ main source of correlated sensorimotor experience for facial actions, but not hand actions, this study provides a litmus test for sensorimotor learning accounts. We presented 4‐month‐old infants with videos of models performing facial actions (e.g. mouth opening, eyebrow raising) and hand actions (e.g. hand opening, finger actions), accompanied by direct or averted gaze, while we measured activation over the corresponding muscle regions using electromyography (EMG) to obtain an index of mimicry. We used EMG because it can reveal sub‐threshold muscle activity that is not visible by eye, and because it has previously been used to investigate the presence of perceptual‐motor couplings in adults (e.g. Fadiga, Craighero, & Olivier, 2005), and, more recently, infants (e.g. Turati, Natale, Bolognini, Senna, Picozzi, Longhi, & Cassia, 2013). The results from the EMG experiment were previously reported in de Klerk, Hamilton, and Southgate (2018). We found that 4‐month‐old infants showed evidence of mimicry when they observed facial actions accompanied by direct gaze but not when they observed facial actions accompanied by averted gaze, and we did not find evidence for mimicry of hand actions^1^ (de Klerk et al., 2018). These results are consistent with previous adult studies that have shown that mimicry effects are enhanced by direct gaze (Bavelas, Black, Lemery, & Mullett, 1986; Postma‐Nilsenová, Brunninkhuis, & Postma, 2013; Wang, Newport, & Hamilton, 2010), and suggest that direct gaze may be a necessary factor to elicit mimicry in infancy. Therefore, this study focussed on the role of sensorimotor experience in supporting infants’ mimicry of actions accompanied by direct gaze (but see the supplementary materials for the equivalent analyses on the averted gaze conditions). After the EMG session, infants participated in a face‐to‐face interaction session with their mother from which we coded the amount of maternal imitation of the same facial actions the infants observed during the EMG session, as well as the amount of time the infant spent looking at their own hands. We hypothesised that maternal imitation of facial actions would be related to the infants’ facial mimicry but not hand mimicry, while the amount of time infants spent looking at their hands would be related to their tendency to mimic hand actions, supporting the idea that sensorimotor experience with actions facilitates the development of the perceptual‐motor couplings that support mimicry behaviour.

## 2 METHODS

### 2.1 Participants

The final sample consisted of 27 infants (*M* = 120 days; range 104–142 days; 10 girls) who were included in the facial EMG analyses and 23 infants (*M* = 120 days; range 104–142 days; 8 girls) who were included in the hand EMG analyses in our previous paper (see Experiment 1 in de Klerk et al., 2018). One additional infant who had good EMG data was excluded from this study because the parent‐child interaction (PCI) session could not be coded due to the positioning of the cameras. All included infants were born full‐term, healthy and with normal birth weight. Written informed consent was obtained from the infant's caregiver prior to the start of the experiment. See the Supplementary materials for more details on exclusion criteria for the EMG analyses, recruitment, and SES.

### 2.2 Procedure

#### 2.2.1 EMG session

**Figure 1:**
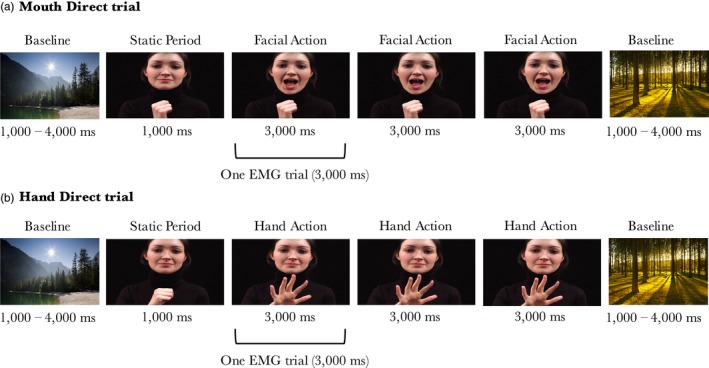
Schematic overview of the stimulus presentation for a mouth direct trial (a) and a hand direct trial (b)

As we use EMG data that was previously analysed, the stimuli and procedure for the EMG experiment are identical to those reported in de Klerk et al. (2018). The EMG experiment took place in a dimly lit and sound attenuated room, with the infant sitting on their parent's lap at approximately 50–60 cm from a 58 cm screen (stimuli subtended a visual angle of approximately 27.2° × 46.0°). Infants were presented with videos of three female models performing eyebrow and mouth actions (e.g. eyebrow raising and mouth opening) and hand actions (e.g. hand opening, finger movements) accompanied by direct or averted gaze, while we measured activation over the eyebrow, mouth, and hand region using EMG. There were six trial types: eyebrow actions accompanied by direct gaze (Eyebrow_Direct), mouth actions accompanied by direct gaze (Mouth_Direct), hand actions accompanied by direct gaze (Hand_Direct), eyebrow actions accompanied by averted gaze (Eyebrow_Averted), mouth actions accompanied by averted gaze (Mouth_Averted) and hand actions accompanied by averted gaze (Hand_Averted). Each video started with 1,000 ms during which the model did not perform any actions, followed by her performing three repeats of the same facial or hand action, each lasting 3,000 ms (see Figure 1). Note that in hand trials the actress did not move her face, and in the face trials the hand was visible but stationary at the bottom of the screen. The 10‐s videos were presented in a random order, alternated with Baseline trials consisting of static pictures of houses, animals, and landscapes with a random duration between 1,000 and 4,000 ms to allow for any mimicry responses to subside before the next video was presented. The session continued until the infant had been presented with approximately 25 videos or until the infant's attention could no longer be attracted to the screen (mean number of presented videos = 26.5, *SD* = 2.8). Infants were video‐recorded throughout the session.

#### 2.2.2 Parent‐child interaction session

After the EMG session infants participated in a 5‐min face‐to‐face interaction session with their mother. For most infants this session took place straight after the EMG session, however, some infants needed a short break for a feed (*N* = 4) or a nap (*N* = 1) between the EMG and PCI session. Infants were placed in a semi‐reclined infant seat facing their mother. Mothers were informed that the researchers would be out of the room for 5 min and that during this time they were to play with their infant however they would at home, when there are no toys around. Three cameras recorded a frontal view of the infant's face, a frontal view of the mother's face, and a side view of the infant's face and body. As some of the infants got fussy towards the end of the PCI session, a total of three minutes of the interaction was video‐coded for the amount of parental imitation (see Section 2.4.1).

### 2.3 EMG recording and processing

Electromyography recording and processing procedures were previously reported in de Klerk et al. (2018). Bipolar EMG recordings were made using paediatric surface Ag/AgCl electrodes that were placed on the cheek (masseter region), forehead (frontalis region) and hand (hand region) with an inter‐electrode spacing of approximately 1 cm. The electrodes were connected to Myon wireless transmitter boxes that amplified the electrical muscle activation, which was in turn recorded using ProEMG at a sampling rate of 2,000 Hz. After recording, the EMG signal was filtered (high‐pass: 30 Hz, low‐pass: 500 Hz) smoothed (root mean square over 20 ms bins), and rectified (converted to absolute values).

Each 3,000 ms period during which a hand or facial action was performed by the model was treated as a separate trial. Videos were coded offline and trials in which the infant did not look at the screen for at least two‐thirds of the action were excluded from analysis.^2^ Additionally, facial action trials during which the infant vocalised, smiled, cried, or had something in their mouth (e.g. their hand or their clothing), and hand action trials during which the infant was moving their arms vigorously or holding onto something, were excluded from the analyses. Only infants with at least three trials per trial type were included in the analyses. On average, the included infants contributed 7.6 trials (*SD* = 3.0) per condition to the analyses. The EMG signal was segmented into 3,000 ms epochs, and the average activity in each epoch was normalised (i.e. expressed as *z*‐scores) within each participant and each muscle group (masseter, frontalis, and hand region), before the epochs for each trial type were averaged together. This allows for meaningful comparison of values between muscle regions, as well as reducing the impact of individual differences in reactivity on the group mean.

As facial mimicry is defined as the presence of greater activation over corresponding muscles than over non‐corresponding muscles during the observation of facial actions (e.g. McIntosh, Reichmann‐Decker, Winkielman, & Wilbarger, 2006; Oberman, Winkielman, & Ramachandran, 2009), we calculated a facial mimicry score per trial by subtracting EMG activity over the non‐corresponding muscle region from EMG activity over the corresponding muscle region (e.g. on an eyebrow trial we subtracted activity over the masseter region from activity over the frontalis region, so that a more positive score indicates more facial mimicry). This resulted in an Eyebrow_Direct mimicry score, a Mouth_Direct mimicry score, an Eyebrow_Averted mimicry score, and a Mouth_Averted mimicry score. We also calculated a mean mimicry score for the Direct and Averted gaze condition. Hand mimicry was measured as the *z*‐scored EMG activity over the hand area during the observation of hand trials, resulting in a Hand_Direct and Hand_Averted mimicry score.

As we only found evidence for mimicry of actions accompanied by direct gaze (de Klerk et al., 2018), we focussed on the role of sensorimotor experience in supporting infants’ mimicry of actions accompanied by direct gaze. However, the equivalent analyses on the averted gaze condition are reported in the Supplementary Materials.

### 2.4 Coding of correlated sensorimotor experience from the PCI

#### 2.4.1 Facial actions

To obtain an index of the infants’ opportunity to associate visual and motor representations of facial actions, we calculated the probability that the mother would copy her infant's facial action within a 3‐s time window. This time window was chosen based on the finding that infants younger than 6 months do not experience events as contingent if they occur more than 3‐s after their own actions (Gergely & Watson, 1999). Videos were coded for the same facial actions that the infants observed during the EMG session (i.e. frowning, eyebrow raising, mouth opening, tongue protrusion) using Mangold INTERACT coding software. To ensure that we would obtain an objective measure of maternal imitation, the videos of the infants and mothers were coded separately ‐ i.e. the coder never played the footage of the infant and the mother simultaneously. We calculated a maternal imitation score by dividing the number of infant facial actions that the mother imitated within 3‐s by the total number of facial actions that the infant performed. The average maternal imitation score was 0.34 (*SD* = 0.14) which is consistent with previous reports (e.g. Moran et al., 1987; Pawlby, 1977; Rayson et al., 2017) ‐ with mothers matching their infants’ actions at a rate of approximately 6 times a minute. As we have a separate eyebrow mimicry score and a mouth mimicry score for our EMG data, we also calculated a separate maternal eyebrow imitation score (*M* = 0.29, *SD* = 0.16) and a maternal mouth imitation score (*M* = 0.40, *SD* = 0.13) to allow us to investigate the specificity of the relationship between maternal imitation and infant facial mimicry.

#### 2.4.2 Hand actions

To obtain an index of the infants’ opportunity to associate visual and motor representations of hand actions, we calculated the proportion of time the infant spent looking at their own hands during the PCI session. The average proportion of time infants spent looking at their own hands was low (*M* = 0.08, *SD* = 0.08), which is not surprising given that this measure was taken during a face‐to‐face interaction with the mother. However, the variability in the proportion of time the infant spent looking at their hands may still provide a useful index of infants’ relative interest in their own hands.

## 3 RESULTS

### 3.1 Facial mimicry

**Figure 2:**
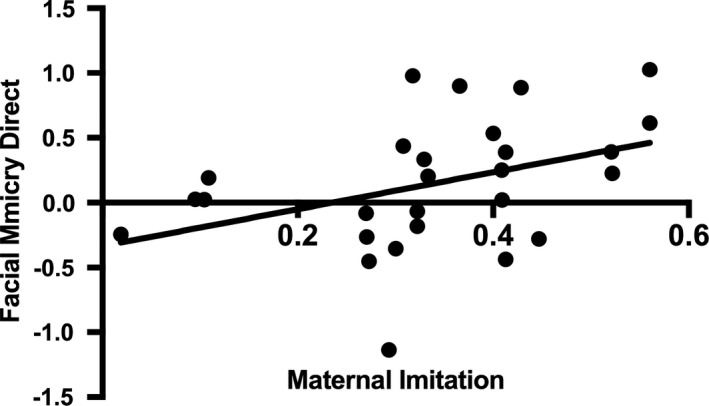
Scatter plot of the relationship between maternal imitation during the PCI and infants’ mean facial mimicry scores in the direct gaze conditions

As predicted, correlational analyses demonstrated that there was a significant relationship between maternal facial imitation and infant mimicry of facial actions accompanied by direct gaze, *r*(25) = 0.392, *p* = 0.043 (lower 95% CI = 0.156, upper 95% CI = 0.614; all confidence intervals were estimated using bootstrapping with 1,000 replication samples) (see Figure 2). Thus, infants whose mother provided them with more opportunities to form associations between visual and motor representation of facial actions showed greater facial mimicry.

**Figure 3:**
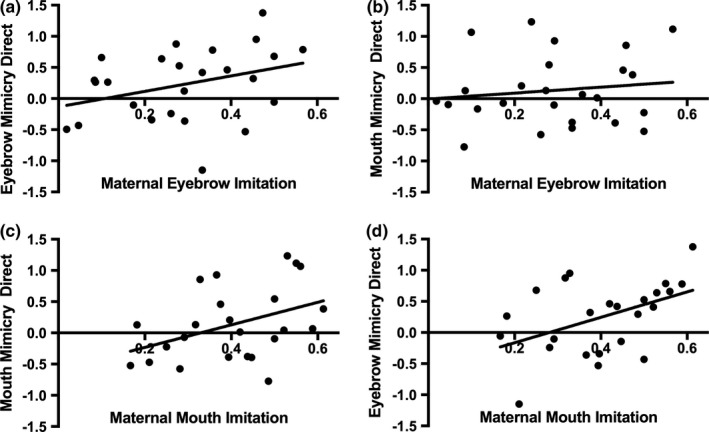
Scatter plots of the relationship between maternal imitation of eyebrow and mouth actions during the PCI and infants’ eyebrow and mouth mimicry scores in the direct gaze condition

Further correlational analyses demonstrated that there was a trend towards a positive correlation between maternal imitation of eyebrow actions and infant eyebrow mimicry, *r*(23) = 0.336, *p* = 0.100 (lower 95% CI = −0.040, upper 95% CI = 0.658) (Figure 3a) while there was no correlation between maternal imitation of eyebrow actions and infant mouth mimicry, *r*(23) = 0.134, *p* = 0.524 (lower 95% CI = −0.278, upper 95% CI = 0.522) (Figure 3b). Maternal imitation of mouth actions was positively correlated with infant mouth mimicry, *r*(22) = 0.407, *p* = 0.049 (lower 95% CI = 0.039, upper 95% CI = 0.659) (Figure 3c) and infant eyebrow mimicry, *r*(22) = 0.458, *p* = 0.024 (lower 95% CI = 0.057, upper 95% CI = 0.751) (Figure 3d). These data thus only provide tentative evidence for the specificity of the relationship between maternal facial imitation and infant facial mimicry.

**Figure 4:**
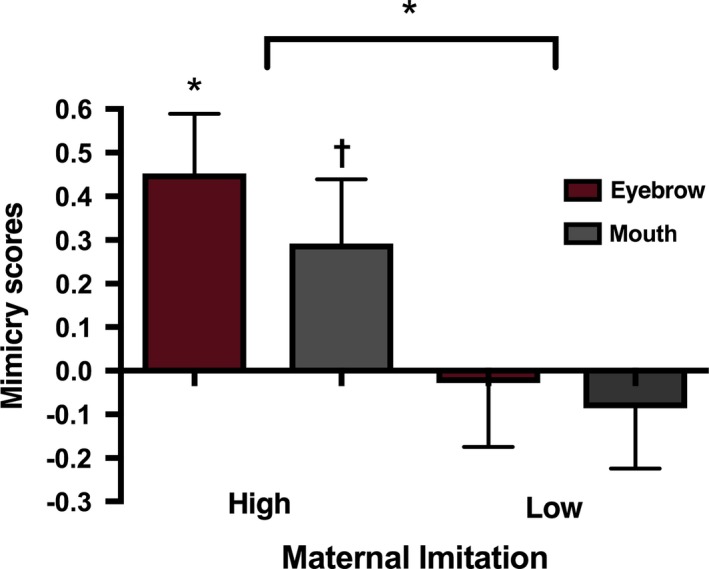
Mean mimicry scores (activation over the corresponding muscle region minus activation over the non‐corresponding muscle region) during the observation of eyebrow and mouth actions accompanied by direct gaze in the high and low maternal facial imitation groups. *p < 0.05, ^†^0.05 < *p* < 0.1. Error bars indicate 1 SEM

We also created a grouping variable based on a median split of the mean maternal facial imitation score, resulting in a high (*N* = 14) and low (*N* = 13) maternal facial imitation group, to investigate the facial mimicry responses in those infants who receive relatively high and low levels of maternal imitation. When we included this variable as a between‐subjects factor in a repeated measures analysis on the mimicry scores in the direct gaze condition with Action type (Eyebrow vs. Mouth) as within‐subjects factors, we found a significant main effect of maternal imitation group, *F*(1, 25) = 6.617, *p* = 0.016, η_*p*_^2^ = 0.209. As can be seen in Figure 4, only those infants in the high‐maternal imitation group showed evidence of facial mimicry, while those in the low‐maternal imitation group did not (see the Supplementary Materials for the equivalent analyses on the *z*‐scored EMG activity per muscle region, and for the equivalent analyses on the averted gaze condition).

### 3.2 Hand mimicry

**Figure 5:**
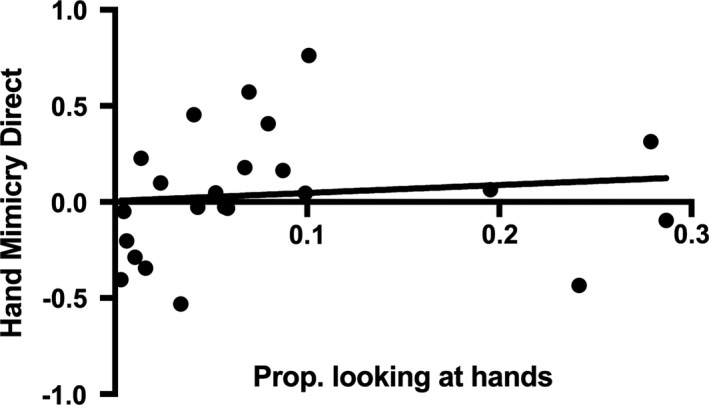
Scatter plot of the relationship between the proportion of time the infant spent looking at their own hands during the PCI and the infants’ hand mimicry scores in the direct gaze condition

**Figure 6:**
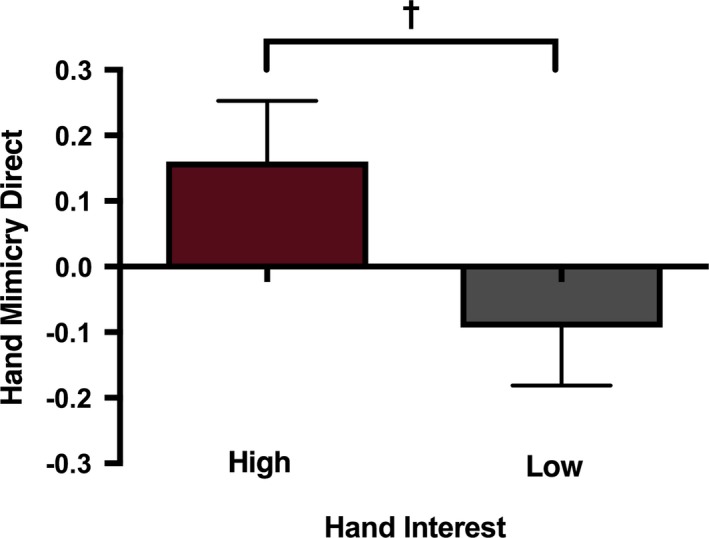
Mean EMG activity over the hand area (hand mimicry) during the observation of hand actions accompanied by direct gaze in the high and low hand interest groups. ^†^0.05 < *p* < 0.1. Error bars indicate 1 SEM

Correlational analyses demonstrated that there was no significant relationship between the proportion of time the infants spent looking at their own hands during the PCI and their mimicry of hand actions accompanied by direct gaze, *r*(21) = 0.108, *p* = 0.623 (lower 95% CI = −0.269, upper 95% CI = 0.547) (see Figure 5). However, as can be seen in Figure 5 the absence of an effect may have been driven by the presence of several influential data points on the right hand side of the scatterplot, i.e. infants who showed a high interest in their own hands during the PCI but who had low hand mimicry scores. To curb the impact of these influential points, we created a grouping variable based on a median split of the proportion of time the infants spent looking at their own hands, to investigate the hand mimicry responses in those infants who showed a relatively high and low level of interest in their own hands. An ANOVA on the hand mimicry in the direct gaze condition with “hand interest” group (high vs. low) as between‐subjects factor showed a marginally significant effect of group, *F*(1, 21) = 3.855, *p* = 0.063, η_*p*_^2^ = 0.115. As can be seen in Figure 6, infants in the high hand interest group (*N* = 12) showed a greater tendency to mimic hand actions compared to infants in the low hand interest group (*N* = 11) (see the Supplementary Materials for the equivalent analyses on the averted gaze condition).

Crucially, when we used the maternal facial imitation grouping variable as a between‐subjects factor, we found that there was no difference in hand mimicry between infants in the high versus low maternal imitation group, *F*(1, 21) = 0.095, *p* = 0.761, η_*p*_^2^ = 0.004.

Additionally, maternal facial imitation was not positively correlated with infant hand mimicry, *r*(21) = −0.350, *p* = 0.102 (lower 95% CI = −0.625, upper 95% CI = −0.014). Thus it does not seem to be the case that infants of high mimicking mothers show greater hand mimicry as well.

Previously we did not find evidence for mimicry of hand actions as measured by EMG, which may raise concerns about the reliability of the hand EMG measure (de Klerk et al., 2018). Therefore, to ensure that the effects described above were reliable, we also coded the videos of the EMG sessions for overt mimicry of hand actions and performed the same analyses on these overt hand mimicry scores. The results replicated those on the hand mimicry as measured by EMG, with infants in the high hand interest group demonstrating a greater tendency to overtly mimic hand actions accompanied by direct gaze compared to infants in the low hand interest group (see Supplementary Materials), providing converging evidence for the idea that infants’ interest in their own hands may be related to their tendency to mimic others’ hand actions.

## 4 DISCUSSION

This study investigated whether the extent to which an observed action elicits mimicry depends on the opportunity the infant has had to develop perceptual‐motor couplings for this action through correlated sensorimotor experience. We found that mothers’ tendency to copy their infants’ facial actions was related to infants’ facial mimicry, while we found preliminary evidence that infants’ tendency to look at their own hands may be related to their tendency to mimic hand actions. These findings are consistent with a recent study by Rayson et al. (2017), that showed that maternal facial imitation during a PCI session at 2 months was related to the infants' sensorimotor cortex activation during the observation of facial actions at 9 months. While it is assumed that sensorimotor cortex activation reflects the existence of perceptual‐motor couplings which give rise to facial mimicry, the current study shows directly that the opportunity to form perceptual‐motor couplings influences mimicry behaviour. Together, these results provide support for the idea that mimicry is supported by perceptual‐motor couplings that are formed through correlated sensorimotor experience obtained by observing one's own actions and imitative social partners (Heyes, 2001; Ray & Heyes, 2011).

Our findings may also shed light on previous studies that have found a lack of spontaneous facial mimicry in children with autism spectrum disorder (ASD) (Beall, Moody, McIntosh, Hepburn, & Reed, 2008; McIntosh et al., 2006; Oberman et al., 2009; but also see Press, Richardson, & Bird, 2010). Children with ASD show reduced or atypical orienting to social stimuli, including faces, from an early age (Chawarska, Macari, & Shic, 2013; Osterling & Dawson, 1994; Zwaigenbaum, Bryson, Rogers, Roberts, Brian, & Szatmari, 2005). This early risk marker for ASD may limit the opportunities these infants have to form perceptual‐motor couplings for facial actions, and could consequently lead to a lack of spontaneous facial mimicry later in life. Future work should investigate the amount of correlated sensorimotor experience with facial actions infants at risk for ASD receive, and how this might relate to later diagnostic outcomes and spontaneous facial mimicry behaviours.

We did not find any positive relationships between maternal facial imitation and infants’ mimicry of hand actions, and instead we found evidence to suggest that infants’ interest in their own hands may be related to hand mimicry. This suggests that it is not the case that infants of high mimicking mothers show greater mimicry overall, which could potentially reflect a more general enhanced prosocial attitude or a greater attention to others’ actions driven by heritable factors (e.g. Hughes & Cutting, 1999; Scourfield, Martin, Lewis, & McGuffin, 1999), but rather that infants specifically mimicked those actions that they received correlated sensorimotor experience with. Nevertheless, while we found a trend towards maternal imitation of eyebrow actions being specifically correlated with the infants’ eyebrow mimicry, maternal imitation of mouth actions was positively correlated both with infants’ mouth and eyebrow mimicry. Thus our study only provides tentative evidence for the specificity of the relationship between sensorimotor experience with an action and the tendency to mimic that action. Potentially, while the 3‐min PCI session provided an accurate representation of the mothers’ overall tendency to copy their infants’ facial actions, there may not always have been enough instances of eyebrow and mouth actions to obtain a representative index of the mothers’ tendency to copy eyebrow and mouth actions specifically. Future studies in which the sensorimotor experience with actions is systematically manipulated are needed to unequivocally determine whether correlated sensorimotor experience with a specific action indeed plays a critical role in supporting mimicry of that action.

Finally, although our findings are consistent with the sensorimotor learning accounts, they cannot resolve the debate surrounding newborn imitation. Even though we found that infants’ facial mimicry was related to the amount of sensorimotor experience they received with these facial actions, this does not preclude the possibility that some rudimentary ability to mimic facial actions is also present from birth (Simpson et al., 2014). Nevertheless, given that one would expect adaptive, inborn mechanisms to be robust against perturbations resulting from naturally occurring variations in the environment (Heyes, 2010), these findings add to the increasing support for the idea that sensorimotor experience may be necessary for the formation of perceptual‐motor couplings that support mimicry behaviour (see also McKyton, Ben‐Zion, & Zohary, 2018).

## CONFLICT OF INTEREST

The authors declare that there is no conflict of interest.

## Endnotes

1 Note that although we did not find evidence for mimicry of hand actions at the group level, this does not preclude the possibility that there is nevertheless meaningful variability in the EMG measure of hand mimicry. However, to mitigate any potential concerns about the reliability of the hand EMG measure we also coded the videos of the EMG sessions for overt hand mimicry and reported the analyses performed on these measures in the Supplementary Materials.

2 Note that it was straightforward to judge whether infants attended the action, as the actions were very prominent on the screen. In most cases this was a binary decision, i.e. the infant either did or did not look at the action on the screen. If the infant made short glances away from the screen, frame‐by‐frame videocoding of the infant's direction of attention was used to determine whether they had seen at least two‐thirds of the action.
