# Supplementary material for: The role of sensorimotor experience in the development of mimicry in infancy
Source: Dev Sci. 2018 Dec 4;22(3):e12771. doi: 10.1111/desc.12771 (PMC6767077; doi:10.1111/desc.12771)
Supplement: Supplementary file 1 [file DESC-22-na-s001.pdf]

Supplementary materials

S.1. Participant information

32 infants were excluded from the original facial EMG analyses due to technical error (N = 3), or because they did not provide enough trials for analyses due to: fussiness (N=10), inattentiveness (N =12), because they constantly vocalised or repeatedly put their fingers in their mouth (N = 5), or because they had raised eyebrows throughout the experiment (N =2). An additional five infants were excluded from the hand EMG analyses due to: technical error (lost EMG signal from the hand EMG transmitter box) (N=1), because they were holding on to their mother's hands for the entire session (N=3), or because their hands were not visible in the video recording for a sufficient number of trials (N=1).

Infants were recruited from a database of parents who voluntarily signed up to participate in infant studies with their child. No direct information on socioeconomic status (SES) was obtained, however, maternal education level (as a proxy for SES) is reported here. Of the included infants, 48 % of the mothers had an undergraduate degree or higher national diploma (HND), the remaining 52 % had a postgraduate degree or doctorate.

S.2. Individual muscle activations

In the paper we reported an effect of maternal imitation group on the mimicry scores in the direct gaze condition. Here we demonstrate that the results are identical when we analyse the individual muscle activations instead. A repeated measures analysis on the EMG activity with Muscle region (Frontalis vs. Masseter), and Action type (Eyebrow vs. Mouth) as within-subjects factors, and maternal imitation (high vs. low) as between-subjects factor demonstrated a significant interaction between Muscle region, Action, and Maternal imitation, $F(1, 25) = 6.617$, $p = .016$, $\eta_p^2 = .209$. We followed-up on the significant three-way

interaction by performing separate repeated measures analyses for the high and low maternal imitation group with Muscle region (Frontalis vs. Masseter), and Action type (Eyebrow vs. Mouth) as within-subjects factors. These analyses demonstrated that there only was a significant interaction between Muscle and Action type in the high maternal imitation group, $F(1, 13) = 10.335$, $p = .007$, $\eta_p^2 = .443$, but not in the low maternal imitation group, $F(1, 12) = .224$, $p = .645$, $\eta_p^2 = .018$. Infants in the high maternal imitation group showed significantly more frontalis region activation than masseter region activation during the observation of eyebrow actions, $t(13)=3.297$, $p=.006$, significantly more frontalis region activation during the observation of eyebrow actions than during the observation of mouth actions, $t(13)=3.228$, $p=.007$, and marginally significantly more masseter region activation than frontalis region activation during the observation of mouth actions, $t(13)=1.987$, $p=.068$ (See Supplementary Figure 1a). Additionally, only in the high maternal imitation group was the EMG activity over the frontalis region during the observation of eyebrow actions significantly different from zero, $t(13)=3.227$, $p=.007$. Thus, only in the high maternal imitation group was there evidence for mimicry, in particular over the eyebrow region.

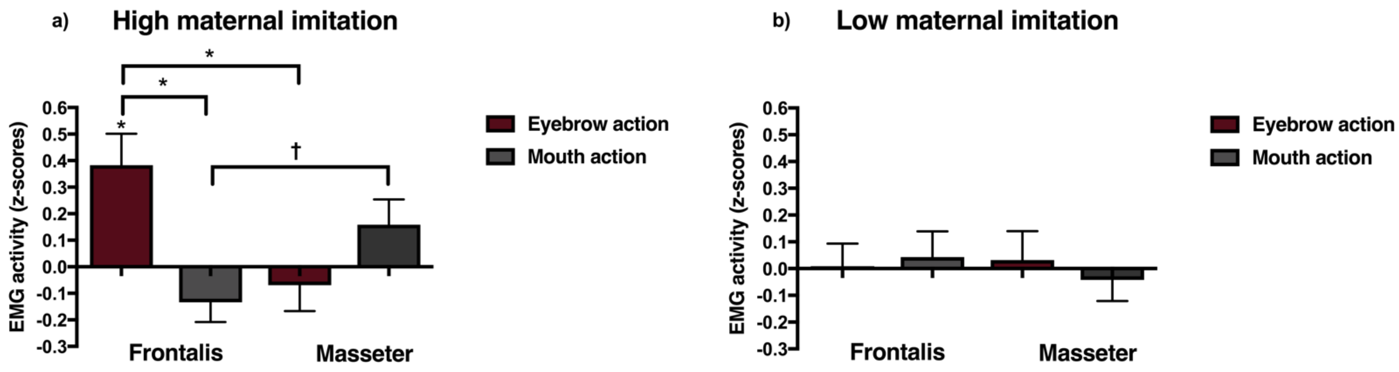

Supplementary Figure 1. a) Mean EMG-activity (z-scores) over the frontalis region and masseter region during the observation of eyebrow and mouth actions accompanied by direct gaze in the high maternal imitation group. * $p < .05$. Error bars indicate 1 SEM. b) Mean EMG-

activity (z-scores) over the frontalis region and masseter region during the observation of eyebrow and mouth actions accompanied by direct gaze in the low maternal imitation group. * $p < .05$. Error bars indicate 1 SEM.

S.3. Averted gaze condition

In the paper we focused on the relationship between sensorimotor experience and mimicry of actions accompanied by direct gaze, as this was the only condition in which we previously found evidence for mimicry (de Klerk et al., 2018). For completeness, we report the results for the equivalent analyses performed on the averted gaze conditions here.

S.3.1. Facial mimicry

Correlational analyses demonstrated that there was no significant relationship between maternal facial imitation and infant mimicry of facial actions accompanied by averted gaze, $r(25)=.021$, $p=.917$ (lower 95% CI= -.432, upper 95% CI = .389) (See Supplementary Figure 2). When we included the maternal facial imitation grouping variable as a between-subjects factor in a repeated measures analysis on the mimicry scores in the averted gaze condition with Action type (Eyebrow vs. Mouth) as within-subjects factors, we found no significant main effects or interactions, all p's > .720. Thus, we did not find evidence for a relationship between maternal imitation and infant mimicry of facial actions accompanied by averted gaze.

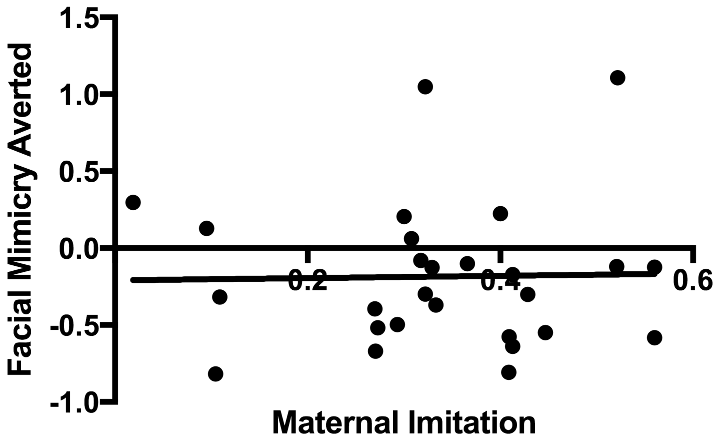

Supplementary Figure 2. Scatter plot of the relationship between maternal facial imitation during the PCI and infants' mean facial mimicry scores in the averted gaze condition.

S.3.2. Hand mimicry

There was no significant relationship between the proportion of time the infants spent looking at their own hands during the PCI and their mimicry of hand actions accompanied by averted gaze, r (21) = -.054, p =.806 (lower 95% CI=-.507, upper 95% CI = .291) (See Supplementary Figure 3). When we included the hand interest grouping variable as a between-subjects factor in an ANOVA on the hand mimicry scores in the averted gaze condition, we found a marginally significant effect of group, F (1, 21) = 3.493, p = .076, η_p^2 = .143. As can be seen in Supplementary Figure 4, infants in the high hand interest group (N=12) showed a diminished tendency to mimic hand actions accompanied by averted gaze compared to infants in the low hand interest group (N=11).

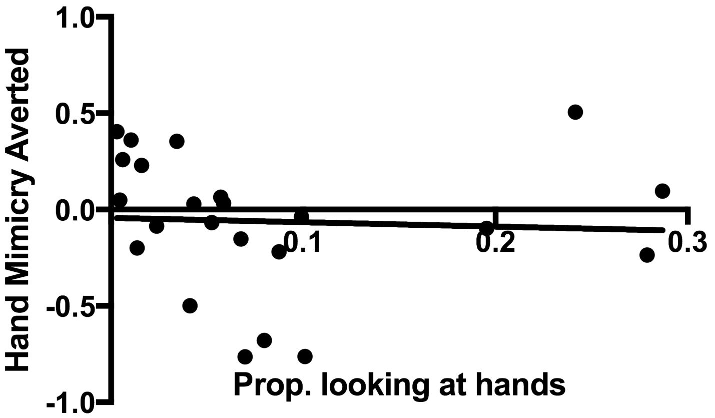

Supplementary Figure 3. Scatter plot of the relationship between the proportion of time the infant spent looking at their own hands during the PCI and the infants' hand mimicry scores in the averted gaze condition.

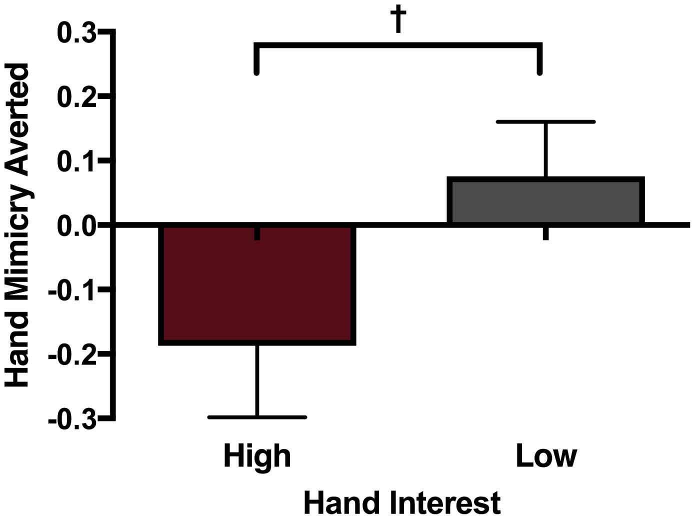

Supplementary Figure 4. Mean EMG activity over the hand area (Hand Mimicry) during the observation of hand actions accompanied by averted gaze in the high and low hand interest groups. † $.05 < p < .1$. Error bars indicate 1 SEM.

S.4 Overt hand mimicry coding

Previously we did not find evidence for mimicry of hand actions (de Klerk et al., 2018). Although this does not preclude the possibility that there is nevertheless meaningful variability in the EMG measure of hand mimicry, it is also possible that the hand EMG data

was not reliable enough to detect an effect (for example because the electrodes were too big to measure from specific hand muscles). Therefore, to obtain an additional index of hand mimicry we also coded the videos for overt mimicry of hand actions. Videos were coded offline and all trials in which the infant performed hand actions such as hand opening and closing, or finger movements, were given a code of 1. Trials in which the infant did not see at least two thirds of the action were excluded from analysis. Additionally, trials were excluded if the infant's hands were not visible in the video or if the infant was holding onto something. The coder was unaware of which trial type the infant was observing. We calculated a hand mimicry score by taking the probability that the infant performed a hand action when they observed a hand trial, and subtracting the probability that the infant performed a hand action when they observed a facial action trial. The resulting measure represents the infant's tendency to specifically perform hand actions when observing hand trials in the Hand_Direct and Hand_Averted trials. For three of the infants who were included in the hand EMG analyses their hands were not visible for a sufficient number of trials to code for overt hand mimicry. Thus the analyses on the overt hand mimicry were based on 20 infants.

S.4.1 Overt hand mimicry results

The results of the analyses on the overt hand mimicry scores replicated those on the EMG measures of hand mimicry. There was no significant relationship between the proportion of time the infants spent looking at their own hands during the PCI and their overt mimicry of hand actions accompanied by direct or averted gaze, all p's > .756. However, again when we created a grouping variable based on a median split of the proportion of time the infants spent looking at their own hands, we found group differences in overt hand mimicry in the direct gaze condition between those infants who showed more versus less interest in their own hands during the PCI. An ANOVA on the overt hand

mimicry scores in the direct gaze condition with 'hand interest' group (High vs. Low) as between subjects' factor showed a marginally significant effect of group, $F(1, 18) = 3.847$, $p = .065$, $\eta_p^2 = .176$. There was no effect of hand interest group on overt mimicry of hand actions accompanied by averted gaze, $F(1, 18) = .002$, $p = .966$, $\eta_p^2 = .00$. As can be seen in Supplementary Figure 5, infants in the high hand interest group (N=10) showed a greater tendency to overtly mimic hand actions accompanied by direct gaze compared to infants in the low hand interest group (N=10).

Again, there was no effect of maternal facial imitation group on overt hand mimicry, $F(1, 18) = .783$, $p = .388$, $\eta_p^2 = .042$, and no correlation between maternal facial imitation and infant overt hand mimicry, $r(18) = -.037$, $p = .878$ (lower 95% CI= -.361, upper 95% CI = .270). The fact that these results are the same as those obtained using the hand EMG measure suggests that this latter measure provided a reliable index of mimicry, and provides converging evidence for the idea that infants' interest in their own hands seems to be related to their tendency to mimic others' hand actions.

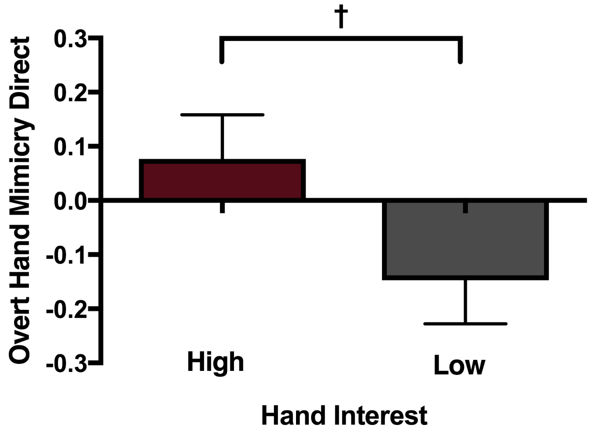

Supplementary Figure 5. Mean overt hand mimicry during the observation of hand actions accompanied by direct gaze in the high and low hand interest groups. † $.05 < p < .1$. Error bars indicate 1 SEM.
